# MRI Findings and Topographic Distribution of Lesions in Metronidazole-Induced Encephalopathy

**DOI:** 10.7759/cureus.29145

**Published:** 2022-09-14

**Authors:** Ambreen Fatima, Sachin Khanduri, Sadaf Sultana, Surbhi ., Saim A Siddiqui, Ashkrit Gupta, Vaibhav Pathak, Mohsin Mulani, Salma Khan, Tanya Bansal

**Affiliations:** 1 Radiodiagnosis, Era’s Lucknow Medical College and Hospital, Lucknow, IND

**Keywords:** brain stem, metronidazole, toxicity, dentate nucleus, corpus callosum, basal ganglia

## Abstract

Purpose

This study aims to describe the magnetic resonance imaging (MRI) of the brain of five patients diagnosed with metronidazole-induced encephalopathy (MIE). In addition, the aim of our study was to better define the topographic distribution of lesions in MIE.

Methods

We retrospectively evaluated MRI findings before and after drug cessation in five patients diagnosed with MIE at Era’s Lucknow Medical College and Hospital, Lucknow, Uttar Pradesh, India. The main MRI signal changes and lesion locations were studied.

Results

Among the patients observed, the average age of the patients with MIE was 55 years (range: 30-70 years). Cerebellar dysfunction, mainly ataxia, and altered mental status were seen in the majority of cases. The most frequently involved sites were the dentate nucleus (cerebellum), brain stem, and corpus callosum (splenium). In diffusion-weighted imaging (DWI), most lesions did not show true restricted diffusion, except for a solitary corpus callosum lesion.

Conclusion

Although drug-related side effects are more common with long-term use of metronidazole, they may also occur with high doses for short durations. The dentate nucleus, the splenium in the corpus callosum, and the brain stem are the most affected structures. Apart from a solitary lesion of the corpus callosum, all identified lesions were reversible at follow-up MRI after discontinuation of metronidazole. The clinical presentation and characteristic MRI changes are highly specific and can be correlated to make a rapid and more accurate diagnosis of this potentially treatable condition. Prognosis is excellent if detected early.

## Introduction

Metronidazole is one of the most widely used antibiotics globally. It is a nitroimidazole derivative that has been clinically used for over three decades and is essential in treating numerous diseases. Metronidazole is efficient against various anaerobic bacteria and protozoan infections affecting the gastrointestinal tract, genitourinary tract, musculoskeletal system, and central nervous system (CNS) [1]. It has high cellular penetration, with the capacity to freely infiltrate the CNS and cerebrospinal fluid (CSF). Metronidazole is usually well tolerated, especially when administered at appropriate doses over a short period. Rare adverse effects, including peripheral neuropathy and neurological dysfunction, may occur at substantial cumulative dosages, particularly if the dosage exceeds 2 g/day for a prolonged period [2]. The neurotoxicity of metronidazole is independent of dosage and intake duration [3].

The term “metronidazole-induced encephalopathy” (MIE) was coined to define the broad spectrum of symptoms due to metronidazole toxicity. The remarkable features of brain magnetic resonance imaging (MRI) are used to diagnose MIE due to the ambiguity of symptoms. MIE patients usually have bilateral symmetric hyperintense signals in T2-weighted images (T2WI) and fluid-attenuated inversion recovery (FLAIR) images of the cerebellar hemispheres, brain stem, and callosal commissure lesions [4]. On follow-up MRI, the lesions usually resolve, as do the symptoms several days after the discontinuation of the drug [5].

Some features such as the pathophysiology of this entity are unclear. However, MIE is exceedingly rare, yet the incidence rate remains unknown. Although it is difficult to predict which patients are most susceptible to toxicity, the likelihood of MIE increases with higher cumulative doses [6]. Despite its rarity, recognizing the characteristic imaging signals associated with MIE may allow clinicians to accurately establish the differential diagnosis of bilateral symmetrical dentate nuclei involvement and expedite the diagnosis [5]. As the pathogenesis of this disease is unknown, the reasons for metronidazole’s predisposition to multiple neuroanatomic structures are still unclear.

## Materials and methods

Study design

After receiving clearance from the Institutional Ethical Committee, we conducted a retrospective study in the Radiodiagnosis Department of Era’s Lucknow Medical College and Hospital, Lucknow, Uttar Pradesh, India, between January 2021 and May 2022.

Inclusion criteria

The study included all patients who met the following clinical criteria for MIE: a recent history of metronidazole intake within three months of having an acute onset of neurological symptoms with characteristic MRI findings.

Exclusion criteria

The exclusion criteria included undocumented medicine intake, atypical or normal imaging findings in MRI, and alternate diagnoses on imaging. Overall, five patients meeting the inclusion criteria were studied. In all patients with clinical suspicion of MIE, metronidazole therapy was discontinued.

MRI protocol and analysis of images

After hospitalization, MRI was performed on all five patients using a 1.5 Tesla MRI system (Signa Explorer, General Electric Healthcare, WI, USA) and a 0.4 Tesla MRI system (Aperto, Hitachi, Japan). T1-weighted images (T1WI), T2WI, diffusion-weighted imaging (DWI), and FLAIR images were acquired in axial planes. The focus of the study was to analyze the distribution of lesions, patterns of diffusion restriction, and reversal of lesions on follow-up MRI. Data from electronic medical records were analyzed, including patient demographics, underlying pathology, drug administration period, presenting neurological symptoms, duration of medication use to the onset of symptoms, duration between onset of symptoms and first MRI, symptom resolution following drug withdrawal, and time period between drug withdrawal and follow-up MRI (in three out of five patients).

Retrospective MRIs were evaluated for the anatomic sites of lesions that demonstrated hyperintense signals on T2WI or FLAIR images. On DWI, the signal intensities of the lesions were also assessed. The resolution of brain lesions was reviewed using follow-up MRIs.

## Results

Demographics and clinical presentation from the case series

Table 1 illustrates the clinical data of the five patients with MIE.

**Table 1:** Demographic data and clinical manifestations of MIE in five diagnosed cases from the series. MIE: metronidazole-induced encephalopathy; M: male; F: female; NA: follow-up MRI not available; MRI: magnetic resonance imaging

Case/age (years)/ gender	Underlying pathology	Dosage (g)/duration (days)	Neurological symptoms	Duration of medication use to the onset of symptoms (days)	Onset of symptoms and first MRI (days)	Symptom resolution following drug withdrawal (days)	Time period between drug withdrawal and follow-up MRI (days)
1/46/M	Cerebral abscess, liver cirrhosis	60/40	Weakness of the right upper extremity, slurring of speech	20	4	6	16
2/58/M	Intra-abdominal abscess	40.5/30	Slurring of speech, gait disturbance, visual blurring	18	7	5	NA
3/36/M	Urinary tract infection	39/18	Slurring of speech, confusion, tingling sensation in both extremities	17	5	4	12
4/70/F	Sacral osteomyelitis	72.5/32	Slurring of speech, altered mental status	14	4	7	36
5/65/F	Cholangitis	44.5/24	Slurring of speech, gait disturbance	25	3	6	NA

All patients (three males and two females) were between the age group of 30-70 years, with an average age of 55 years. The mean metronidazole dose was 51.3 g (range: 39-72.5 g) over 29 days (range: 18-40 days). The estimated duration from the start of treatment to the onset of neurological symptoms was 18.8 days (range: 14-25 days). Symptoms of cerebellar dysfunction included slurred speech (n = 5), abnormal gait (n = 2), weakness of the right upper extremity (n = 1), blurred vision (n = 1), tingling sensation in both extremities (n = 1), altered mental status (n = 1), and confusion (n = 1). Reversal of neurological symptoms was noted in all patients within 4-7 days, with an average of 5.6 days after the discontinuation of metronidazole. The mean duration between the onset of symptoms and the first MRI was 4.6 days (range: 3-7 days), with a mean gap of 21.3 days (range: 12-36 days) between the discontinuation of the drug and the follow-up MRI (which was done only in three patients).

Topographical distribution of lesions

**Figure 1:**
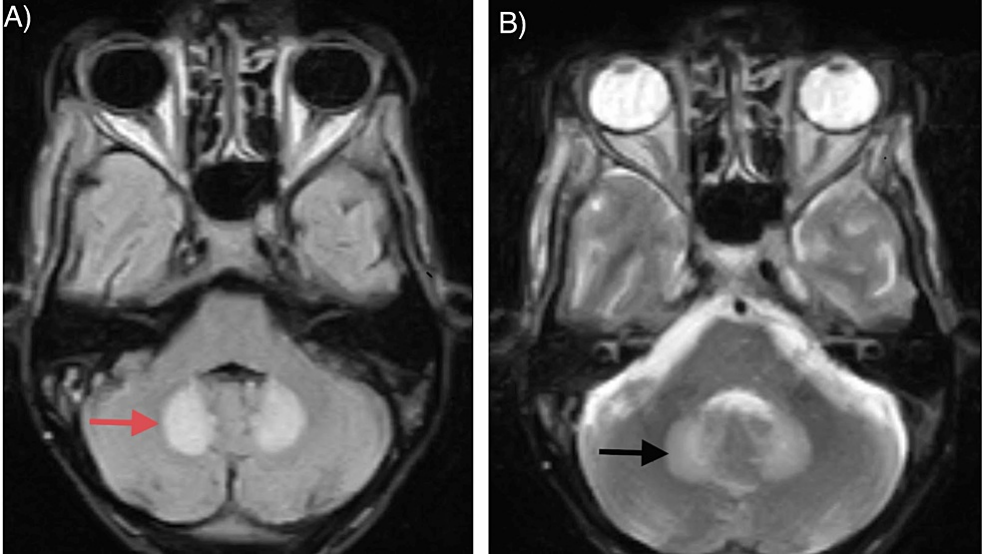
1.5 Tesla MRI of a 58-year-old male with a history of metronidazole intake for pus collection in the abdomen. The (A) axial section of the FLAIR sequence (red arrow) and (B) T2WI (black arrow) demonstrate the characteristics of bilateral symmetric hyperintense lesions in the dentate nuclei of bilateral cerebellar hemispheres. MRI: magnetic resonance imaging; FLAIR: fluid-attenuated inversion recovery; T2WI: T2-weighted image

Hyperintense bilaterally symmetrical lesions were present in the dentate nuclei (n = 5, 100%), midbrain, callosal commissure (corpus callosum), pons, medulla, basal ganglia, thalami, and white matter in the subcortical region of bilateral frontal lobes (Figure 1). Lesions of the midbrain included the tectum (n = 3, 60%), tegmentum surrounding the central gray matter (n = 2, 40%), and red nucleus (n = 1, 20%). Lesions of the dorsal pons involved the vestibular nucleus (n = 2, 40%) and abducens nucleus (n = 2, 40%), and lesions of the caudal part of the pons included the nuclei of the superior olive (n = 1, 20%). There were also lesions in the basal ganglia (n = 2, 40%) and thalami (n = 2, 40%). The nuclei of the inferior olive (n = 1, 20%) and dorsal aspect of the medulla (n = 2, 40%) were the locations of medullary lesions. In patients with pontine and medullary involvement, lesions were consistent in the dorsal aspects of the pons and medulla.

Four of the five patients had corpus callosum lesions involving the splenium. One (20%) patient had involvement of the white matter in the subcortical region of the bilateral frontal lobes. None of the lesions showed enhancement on contrast-enhanced T1WI.

Table 2 depicts topographical lesion distribution on T2WI and FLAIR images.

**Table 2:** Imaging data showing the topographic distribution of lesions on T2WI and FLAIR images in five MIE patients T2WI: T2-weighted image; FLAIR: fluid-attenuated inversion recovery; MIE: metronidazole-induced encephalopathy; CC: corpus callosum; RN: red nucleus; VN: vestibular nucleus; SOC: superior olivary complex (superior olivary nucleus); AN: abducens nucleus; IOC: inferior olivary complex (inferior olivary nucleus); BG: basal ganglia; SWM: subcortical white matter; P: present at each anatomic site; A: absence of abnormality on MRI

Case	Cerebellum	CC	Midbrain			Pons			Medulla		Thalami	BG	SWM
	Dentate nuclei		Tectum	RN	Tegmentum	VN	SOC	AN	Dorsal medulla	IOC			
1	P	P	P	A	A	A	A	P	P	A	A	A	A
2	P	A	P	A	P	P	A	A	A	A	P	P	A
3	P	P	A	A	A	P	P	A	P	A	P	A	A
4	P	P	A	P	A	A	A	A	A	P	A	P	P
5	P	P	P	A	P	A	A	P	A	A	A	A	A

Imaging findings

DWI Signal Intensity

**Figure 2:**
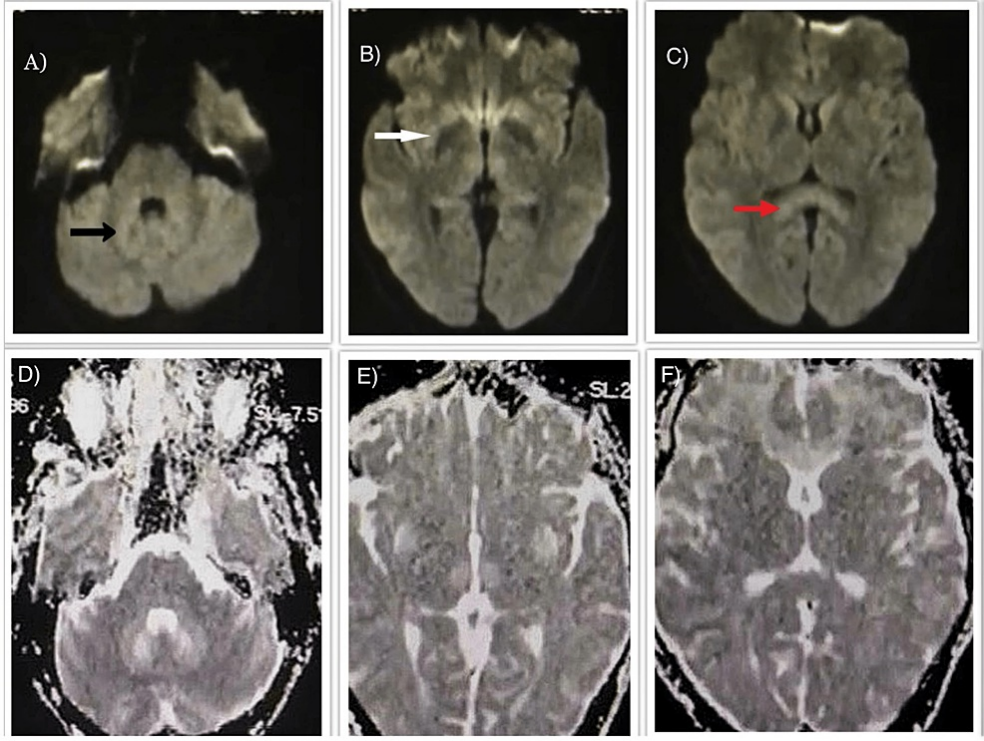
0.4 Tesla MRI and axial DWI reveal hyperintense signals in (A) dentate nuclei (black arrow), (B) basal ganglia (white arrow), and (C) splenium (red arrow). (D-F) ADC maps show high ADC areas in the dentate nuclei and basal ganglia. However, ADC values in these regions were not reduced, meaning there was no true diffusion restriction. MRI: magnetic resonance imaging; DWI: diffusion-weighted imaging; ADC: apparent diffusion coefficient

In bilateral dentate nuclei, mesencephalon, and pontomedullary lesions, DWI signal intensity varied from hyperintense to isointense. Of the four cases involving the corpus callosum, only one showed true diffusion restriction on DWI and apparent diffusion coefficient (ADC). DWI frequently showed increased signals in the bilateral dentate nuclei and various brain stem lesions. However, ADC values were not reduced at these sites (T2 shine-through) (Figure 2).

Lesion Reversibility

**Figure 3:**
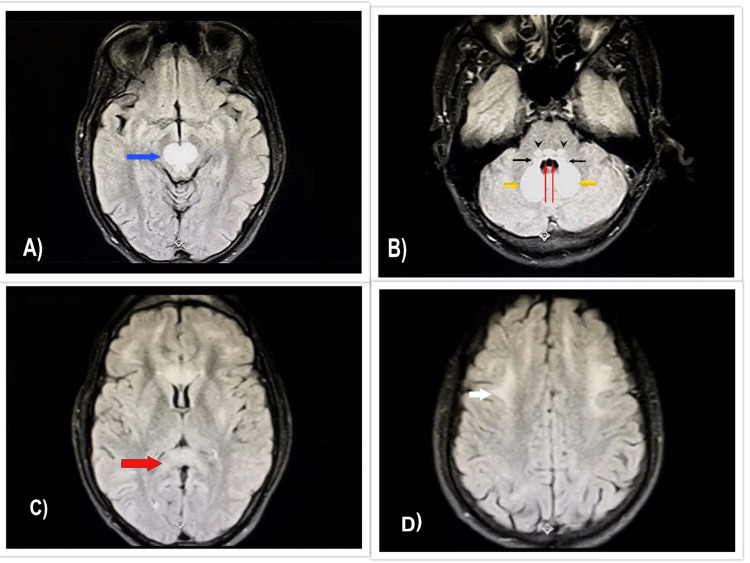
0.4 Tesla MRI and axial FLAIR images demonstrate bilaterally symmetrical hyperintense signals. (A) Midbrain involving red nuclei and tegmentum (blue arrow). (B) Cerebellar dentate nuclei (thick yellow arrows); dorsal pons showing vestibular (thin black arrows) and abducens nuclei (thin red arrows) involvement, while in caudal pons, superior olivary nuclei lesion is seen (arrowheads). (C) Corpus callosum (splenium) (thick red arrow). (D) Frontal lobe subcortical white matter (white arrow). MRI: magnetic resonance imaging; FLAIR: fluid-attenuated inversion recovery

**Figure 4:**
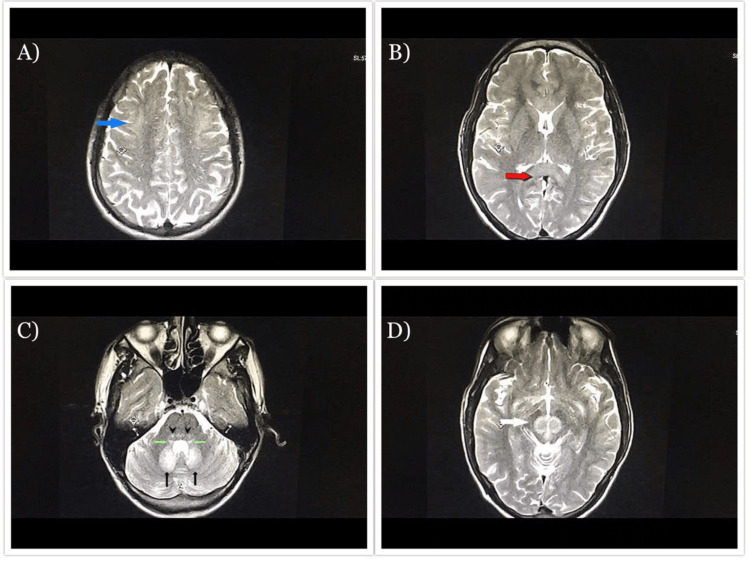
0.4 Tesla MRI and axial T2WI demonstrate a hyperintense signal. (A) Subcortical white matter (blue arrow). (B) Splenium (thick red arrow). (C) Cerebellar dentate nuclei (thin black arrows), vestibular (green arrows), and a superior olivary nuclei lesion of caudal pons (arrowheads). (D) Midbrain involving red nuclei and tegmentum (white arrow). MRI: magnetic resonance imaging; T2WI: T2-weighted image

On follow-up, T2WI and FLAIR images exhibited hyperintense MRI signals that were entirely reversible in two patients; however, they showed a partial reversal in one patient. One of the two patients who underwent follow-up MRI had lesions in the corpus callosum showing localized and persistent hyperintensity during follow-up, mainly in the splenium. In all three patients, lesions apart from those in the corpus callosum had resolved at the follow-up MRI (Figures 3, 4).

## Discussion

**Figure 5:**
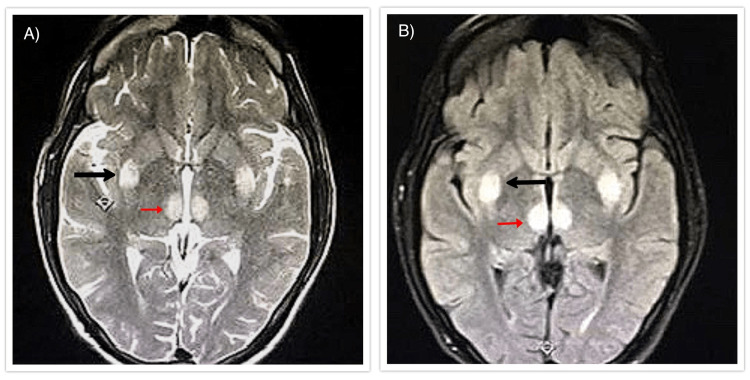
0.4 Tesla MRI demonstrates bilateral symmetric hyperintensity involving bilateral basal ganglia (black arrows) and thalami (red arrows) on axial (A) T2WI and (B) FLAIR. MRI: magnetic resonance imaging; T2WI: T2-weighted image; FLAIR: fluid-attenuated inversion recovery

Three patients underwent 0.4 Tesla MRI, while for the remaining two patients, 1.5 Tesla MRI was used. Our study showed that the dentate nuclei, various sites in the midbrain (tectum, tegmentum around central gray matter, and red nucleus), corpus callosum (splenium), and dorsal parts of the brain stem were the common locations, with bilateral and symmetrical patterns. In addition, the superior and inferior olivary complex, bilateral cerebral hemispheres (subcortical) white matter, basal ganglia, and thalamus were uncommon sites (Figure 5).

As per the already published 10 cases of metronidazole neurotoxicity [7-16], the dentate nuclei are the common sites of involvement on MRI. All cases from our study and nine of the 10 earlier documented cases involved dentate nuclei. The midbrain (n = 7), callosal commissure (n = 7), subcortical white matter (n = 6), basal ganglia (n = 3), and thalami (n = 2) were among the other less common sites in earlier reported patients. Previous studies have also shown the involvement of the dorsal pons (n = 3) and superior olivary nuclei in the caudal part of the pons (n = 1), dorsal part of the medulla (n = 1), and nuclei of the inferior olive (n = 1). In one of these studies, the involvement of the anterior commissure and inferior colliculi of the midbrain was also reported [12,13]. The lesion distribution of MIE in the cumulative dataset of 15 patients from our and earlier documented studies is as follows: dentate nuclei (n = 14, 93%), midbrain (n = 11, 73%), callosal commissure (n = 11, 73%), pons (n = 8, 53%), cerebral subcortical white matter (n = 7, 46%), medulla (n = 5, 33%), basal ganglia (n = 5, 33%), and thalami (n = 4, 26%).

Midbrain lesions had variable structural involvements, which included the tectum, tegmentum, and red nuclei. The vestibular and abducens nuclei in the pontine tegmentum, as well as the superior olivary nucleus in the lower pons, showed a specific pattern of lesions. A T2 hyperintense signal in the vestibular nuclei of the rhomboid fossa at the dorsal medulla and the inferior olivary nuclei was observed in three patients with medullary lesions. Current and earlier studies indicate that MIE patients have identical MRI lesion characteristics and distribution.

First, the lesions of MIE are always bilaterally symmetrical, with the dentate nucleus typically affected in all MIE patients. Second, the classic MRI findings of the pons include lesions in the vestibular nuclei, followed by the abducens nuclei, and the superior olivary nuclei are bilaterally symmetrically distributed. Third, the lesions of the callosal commissure always include the splenium. Only a few instances of inferior olivary nuclei involvement have been documented earlier in MIE. According to Seok et al. [12], the hyperintense signals in the inferior olivary nucleus remained unchanged on MRI follow-up, while they disappeared in the rest of the other lesions. They proposed that lesions disrupting the dentato-rubro-olivary pathway (myoclonic triangle) could cause lesions of the inferior olivary nucleus instead of lesions caused by metronidazole drugs. It is unclear whether hypertrophic olivary degeneration or metronidazole neurotoxicity causes the inferior olivary complex lesions in MIE.

The DWI signal is variable and does not always coincide with a fixed ADC value; in this respect, it is somewhat controversial. Hyperintensity of dentate nuclei, midbrain, or brain stem on T2WI, FLAIR, or DWI combined with an ADC value equal to or greater than the normal white matter strongly suggests vasogenic edema. In contrast, hyperintensity of the corpus callosum on T2WI, FLAIR, and DWI with lower ADC values indicates cytotoxic edema, most likely caused by an ischemic process of axonal fibers [17]. The findings and the existing literature support the view that callosal commissure was the only lesion consistent with low ADC values in many reports. While metronidazole readily crosses the blood-brain barrier and causes vasogenic edema in the dentate nuclei and brain stem, the involvement of the callosal commissure could be a secondary process. The splenium is a common site for cytotoxic edema due to its high glutamate receptor levels. The cytotoxic lesions of the corpus callosum are often associated with multiple phenomena. They are attributed to pharmacological therapy, tumors, septicemia, intracranial hemorrhage, metabolic abnormalities, trauma, and other conditions [18].

Demyelinating diseases and various metabolic, infective, and inflammatory processes are on the list of differential diagnoses of MIE. Acute Wernicke’s encephalopathy is one of the most important MIE differentials. Although MIE and acute Wernicke’s encephalopathy may have similar clinical manifestations, they may be clinically confused in the initial phases. Mammillary bodies, medial thalami, and central midbrain gray matter all show bilaterally symmetric hyperintense signals on T2WI, which are unique to Wernicke’s encephalopathy and not observed in MIE. However, patients with nonalcoholic Wernicke’s encephalopathy may have abnormal imaging features, such as altered T2WI signals in the dentate nuclei, vermis, nuclei of the cranial nerve, and splenium, consistent with MIE [19]. Moreover, in nonalcoholic Wernicke’s encephalopathy, abnormal findings are often accompanied by more specific characteristic features such as ophthalmoplegia. Methyl bromide poisoning is a rare form of neurotoxicity that shows comparable features to MIE on MRI, including T2WI hyperintensities in bilateral symmetrical dentate nuclei, splenium, dorsal aspect of pons and medulla, and cranial nerve nuclei.

Diseases such as multiple sclerosis also lead to lesions of the cerebellum and brain stem. However, the lesions are often asymmetrical and do not directly affect the cranial nerve nuclei. In multiple sclerosis, hyperintense signals on T2WI may be present in the periventricular, cortical, juxtacortical, infratentorial, and brain stem, with active lesions showcasing true restriction diffusion and contrast enhancement [20]. If the splenium shows T2-weighted hyperintensities, other conditions such as Marchiafava-Bignami, encephalitis, epilepsy, extrapontine osmotic demyelination, lupus erythematosus, renal failure, and cobalamin deficiency must be ruled out [21]. Heat stroke affects the thalami and external capsules, whereas cerebellar involvement is rare [22].

In at least 65% of reported cases, there was a complete reversal of symptoms or marked improvement after discontinuation of metronidazole. However, in a few patients (29%), the condition may permanently worsen and lead to death. It is vital to discontinue treatment with metronidazole as soon as possible to avoid deterioration of the patient’s clinical condition [17]. Regardless of the numerous differential diagnoses, toxicity can be reported in all cases with the remarkable topographic pattern of lesions on MRI, i.e., bilateral symmetrical dentate nuclei involvement, and confirmed when there is a classical history of metronidazole therapy present, and the symptoms and MRI features are reversible after discontinuation of treatment. Toxicity due to a drug may be contemplated as a differential diagnosis if multifocal lesions are present with or without mass effect on MRI [22].

Limitations of the study

The current study has some limitations. First, in this study, only five patients (a limited number) were recruited. Second, out of five patients, only three underwent follow-up MRI after symptoms improved. Third, long-term follow-up MRIs for complete lesion resolution were unavailable, making it difficult to determine whether the lesions had resolved entirely in all patients.

## Conclusions

Metronidazole toxicity typically develops in patients who have received high cumulative doses, but it may be dose-independent and manifest after a short duration of treatment. On MRI, MIE brain lesions were always bilateral and symmetrical. T2WI hyperintensity was most pronounced in the dentate nuclei, followed by the midbrain and splenium, in our study. However, pons and medulla, subcortical white matter, basal ganglia, and thalami were less commonly involved. While the vast majority of MIE cases have lesions located at sites of vasogenic edema, only a few lesions located in the corpus callosum are probably areas of cytotoxic swelling.
